# Tumor location impacts immune response in mouse models of colon cancer

**DOI:** 10.18632/oncotarget.18423

**Published:** 2017-06-09

**Authors:** Xianda Zhao, Lihua Li, Timothy K. Starr, Subbaya Subramanian

**Affiliations:** ^1^ Department of Surgery, University of Minnesota Medical School, Minneapolis, MN, USA; ^2^ Department of Obstetrics and Gynecology and Women's Health, University of Minnesota Medical School, Minneapolis, MN, USA; ^3^ Masonic Cancer Center, University of Minnesota, Minneapolis, MN, USA

**Keywords:** colorectal cancer, immune profiles, immunotherapy, mouse models of cancer, orthotopic models

## Abstract

Existing preclinical models of human colorectal cancer (CRC) that rely on syngeneic subcutaneous grafts are problematic, because of increasing evidence that the immune microenvironment in subcutaneous tissue is significantly different from the gastrointestinal tract. Similarly, existing orthotopic models that use a laparotomy for establishing grafts are also problematic, because the surgical procedure results in extensive inflammation, thereby creating a nonphysiologic tumor microenvironment. To facilitate the bench-to-bedside translation of CRC immunotherapy strategies, we developed a novel orthotopic model in mice that uses endoscopy-guided microinjection of syngeneic cancer cells. When we compared immune system infiltration, we found that tumors in the subcutaneous model had fewer T cells, B cells, and natural killer (NK) cells, but more immunosuppressive myeloid cells; in contrast, tumors in our orthotopic model had a higher number of tumor-infiltrating T cells, B cells, and NK cells, with fewer immunosuppressive myeloid cells. The number of immune-stimulating cytokines, such as interleukin (IL)-2, IL-6, interferon (IFN)-gamma, and granzyme B, was also higher in tumors in our model, as compared with the subcutaneous model. Those differences resulted in heightened sensitivity to immune checkpoint blockade therapy in our endoscopy-guided orthotopic CRC model. Our study indicates that tumor location affects immune response in CRC mouse models; choosing the appropriate preclinical model is important when testing immunotherapy in CRC.

## INTRODUCTION

Tumor development in humans is regulated by tumor-specific adaptive immune responses [1]. Recently developed therapies that enhance the immune response, such as chimeric antigen receptor (CAR) T cells and immune checkpoint blockade therapies (ICBTs), have resulted in remarkable outcomes in certain cancers [2, 3]. For example, checkpoint blockade antibodies targeting PD1, PDL1, or CTLA4 have resulted in significantly improved survival in patients with advanced drug-resistant melanoma, lung cancer, and renal cancer [4–6]. However, in patients with colorectal cancer (CRC), which ranks among the most common malignancies in the United States, the efficacy of these therapies was much less remarkable [7].

Preclinical CRC immunotherapy studies have largely depended on syngeneic subcutaneous tumor models [8, 9]. A recent study has shown that tumor location determines tissue-specific recruitment of tumor-associated macrophages in melanoma model [10]. Therefore, the classic subcutaneous models may not mimic the immune tumor microenvironment of actual human CRC and may be a major barrier in efforts to translate findings on immunotherapy from the laboratory to the clinic [11]. Moreover, two immune-infiltrated subtypes of CRC have been seen in human patients: the well-infiltrated subtype and the poorly infiltrated subtype [12, 13]. However, the current subcutaneous CRC models are poorly infiltrated [14]. Therefore, it is critical to establish a preclinical model that recapitulate primary tumor microenvironment and mimic immune well-infiltrated human CRC features, especially their baseline immune response. Such a model would be complementary to the classic subcutaneous models and would facilitate investigation of the mechanisms of ICBT resistance in various clinical settings [15].

Orthotopic tumor models have several advantages over chemically- or genetically-induced tumor models. In general, orthotopic transplants are easier and faster to establish, and they are located within a physiological and microenvironment comparable to that of human diseases [16]. In CRC, orthotopic tumor models have usually required a laparotomy, which can cause a strong inflammatory response, which is a potential confounding factor of experimental outcomes, making it unsuitable for immunotherapy studies [17]. We have established a novel orthotopic CRC model that uses endoscopy-guided microinjection to establish orthotopic tumors in the colon wall in mice [18]. Our minimally invasive CRC model does not provoke an inflammatory response and is particularly suitable for immunotherapy studies. In this study, we investigated the key characteristics of our model, including immune infiltration and responses to ICBT. We compared orthotopic tumors with subcutaneous tumors established from the same CRC cell lines and demonstrate a significant difference in immune response. These findings highlight that tumor location influences immune responses in CRC animal models and the importance of model selection in preclinical immunotherapy studies.

## RESULTS

### Establishment of orthotopic colorectal tumors in mouse

To establish a standard procedure of tumor cell implantation in the mouse colon wall, we tested different anesthesia options. We found that colon spasms and colonic secretion, in response to endoscopic examination or needle puncture, were the most common issues leading to implantation failure. The frequency and degree of colon spasms and colonic secretion were higher in mice anesthetized with Avertin; those issues were controlled when we administered a combination of ketamine, xylazine, and atropine. During the process of tumor cell implantation, a positive lifting of the colonic mucosa at the implantation sites indicated successful cell inoculation (Supplementary Video 1). We have achieved 80% success rate in implanting orthotopic mouse CRC tumors.

**Figure 1:**
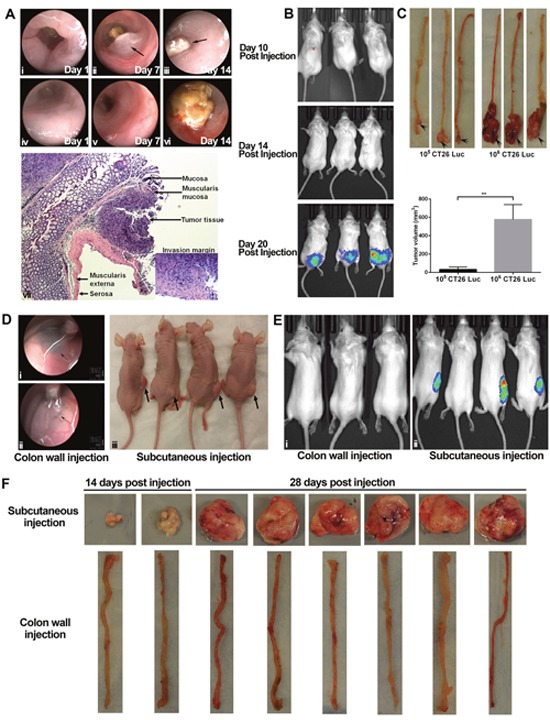
Tumorigenesis in orthotopic and subcutaneous models was distinct Orthotopic tumors were established by endoscopy-guide microinjection in the colon wall of BABL/c mice. In some mice, tumors and tumor-caused colon obstruction can be seen directly in the colon lumen **(A, i-iii)**. In other mice, abnormal movement, stiffness, and bleeding can be seen in the colon **(A, iv-vi)**. H&E staining showed tumor tissue in the submucosa layer. The invasion margin can be seen in the muscularis externa **(A, vii)**. An in *vivo* imaging system can be used for monitoring in our orthotopic model **(B)**. Injection of different numbers of CT26 cells (10^5^ and 10^6^) led to a significance difference in tumor volume at autopsy **(C)**. At 4 weeks after injection of 10^3^ HT29 tumor cells in either the colon wall or the subcutaneous connective tissue of athymic nude mice (n = 4 in each model), tumorigenesis occurred only in the subcutaneous model **(D)**. Arrows indicate the representative injection sites in the colon wall, but no tumors formed **(D, i-ii)**. At 4 weeks after injection of 10^4^ CT26 cells in either the colon wall (**E, i**; n = 3) or the subcutaneous connective tissue (**E, ii**; n = 4) of BALB/c mice, subcutaneous tumors formed **(E, i)**; however, no orthotopic tumors formed **(E, ii)**. Autopsy confirmed the results of imaging (data not shown) **(E)**. Injection of 10^5^ MC38 did not induce tumor formation in the colon wall (n = 8) of C57/B6 mice, whereas it did cause subcutaneous tumor formation (n = 8) **(F)**. H&E: hematoxylin and eosin. **P < 0.01.

When we used endoscopy to monitor tumor growth, we saw abnormal protrusions in the colon lumen around 1 week after injection in some mice (Figure 1A, i-ii); subsequently, rapid tumor growth induced colonic obstruction (Figure 1A, iii). In other mice, as tumors invaded the submucosal layer and expanded toward the serosa, we could not detect tumor growth by bright-light endoscopy; however, we did see stiffness, brittleness, and heavy bleeding in the mucosa (Figure 1A, iv-vi). Histologic analysis indicated that orthotopic tumors were growing in the submucosa layer and invading the muscularis layer (Figure 1A, vii). We detected orthotopic tumors around 3 weeks after implantation of 10^5^ CT26-Luc cells (Figure 1B) by IVIS. A direct correlation between tumor volume and the number of injected tumor cells can be found in our orthotopic model (Figure 1C).

### Fewer cells are required to establish subcutaneous models compared to orthotopic models

In athymic nude mice, orthotopic injection of 10^3^ HT29 cells could induce tumor formation at 4 weeks in the subcutaneous model, but not in orthotopic model (Figure 1D). Orthotopic injection of 10^5^ HT29 cells was sufficient to induce tumor formation and invasion at 4 weeks in the orthotopic model (Supplementary Figure 1A). Similar results were observed in our two syngeneic orthotopic CRC models. For example, injection of 10^4^ CT26 cells induced tumor formation at 4 weeks in subcutaneous tissue, but not in the colon wall in BALB/c mice (Figure 1E; results confirmed by autopsy, data not shown) and injection of 10^5^ MC38 cells induced tumor formation at 4 weeks in subcutaneous tissue, but not in the colon wall in C57BL/6 mice (Figure 1F). Taken together, our data indicate that more cells are required to initiate tumorigenesis in the orthotopic model compared to subcutaneous model.

### Orthotopic tumors have more adaptive immune-cell infiltration than subcutaneous tumors

**Figure 2:**
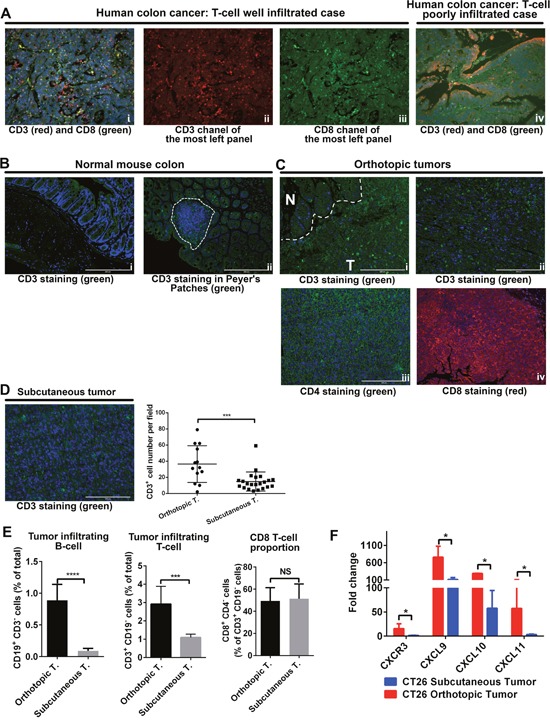
Adaptive immune cell infiltration was higher in orthotopic tumors than in subcutaneous tumors In human CRC samples, both well-infiltrated **(A, i-iii)** and poorly-infiltrated **(A, iv)** tumors were found. In normal BALB/c mouse colon, CD3^+^ cells were usually seen in the mucosa lymph tissues **(B)**. In orthotopic tumors, CD3^+^ cells were seen in the tumor margins **(C, i)** and in the central parts of the tumor **(C, ii)**. Both CD4^+^ and CD8^+^ cells were seen in orthotopic tumors **(C, iii-iv)**. In subcutaneous tumors, CD3^+^ cells were also detected, but not as many as in orthotopic tumors **(D)**. Flow cytometry analysis indicated more B cells and more T cells in orthotopic tumors than in subcutaneous tumors. The proportion of CD8^+^ T cells was the same in the two models **(E)**. In orthotopic tumors, mRNA expression of CXCR3, CXCL9, CXCL10, and CXCL11 was higher. Expression level was presented as fold change, refers to the lowest expression **(F)**. N: normal; T: tumor. (% total refers to total number of cells in tumor tissue) ***P < 0.001; ****P < 0.0001.

Multiple studies have demonstrated that immune cell infiltration in CRC is highly variable between patients and increased infiltration of immune cells was associated with a better outcomes [19]. Immunostaining with T-cell markers (CD3 and CD8) in human CRC samples, we also observed two CRC subtypes, based on T-cell infiltration: *well-infiltrated* tumors (Figure 2A, i-iii) and *poorly-infiltrated* tumors (Figure 2A, iv). In samples of normal colon from BALB/c mice, the microenvironment that orthotopic tumors grew in, we found very few T cells except in the Peyer patches (Figure 2B). This observation may be reflective of the lacks of antigen exposure in these mice and their controlled housing environment [20]. In the orthotopic tumor tissue, we found T cells in both the margins (Figure 2C, i) and the central parts (Figure 2C, ii) of tumors; we saw both CD4^+^ (Figure 2C, iii) and CD8+ cells (Figure 2C, iv). In contrast, in subcutaneous tumors established by the same cell line, we found a very small number of T cells (Figure 2D). Flow cytometry analysis showed the same trends of T-cell infiltration in both models. We then determined the proportion of CD8+ T cells in these two models and found no differences. Since B cells are important in antigen presentation and adaptive immunity. We checked B-cell number in both these models and found the number of tumor-infiltrating B cells was higher in orthotopic tumors than in subcutaneous tumors (Figure 2E). RT-qPCR analysis showed expression of chemokines related to T-cell migration was higher in orthotopic tumors than in subcutaneous tumors (Figure 2F).

### NK cells increased and myeloid-derived suppressive cells are decreased in orthotopic tumors

**Figure 3:**
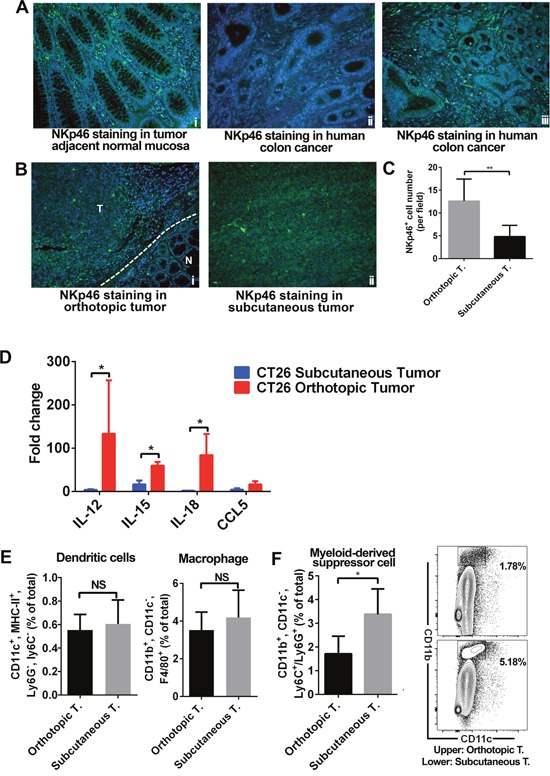
Innate immune cell infiltration in orthotopic and subcutaneous models was distinct In human colon samples, NKp46^+^ cells were found in normal colon and in some CRC cases **(A)**. In tumor models, NKp46^+^ cells were more frequent in orthotopic tumors than in subcutaneous tumors **(B-C)**. mRNA expression of IL-12, IL-15, IL-18, and CCL5 (all related to NK cells) was higher in orthotopic tumors. Expression level was presented as fold change, refers to the lowest expression **(D)**. Flow cytometry showed no difference in the number of DCs and macrophages in the two models **(E)**. However, more MDSCs, important immunosuppressive cells, were found in subcutaneous tumors **(F)**. A higher proportion of CD11b^+^ CD11c^-^ cells were found in subcutaneous tumors **(F)**. The proportion of Ly6C^+^ and Ly6G^+^ cells (in the CD11b^+^ CD11c^-^ population) was the same in the two models (data not shown). IL: interleukin; N: normal; T: tumor. (% total refers to total number of cells in tumor tissue) *P < 0.05.

Innate immune cells are also critical in regulating antitumor immune responses. Therefore we compared the innate immune profiles between these two models. We observed high levels of NKp46^+^ cells in human normal colon tissues (Figure 3Ai). However, in CRC patient samples, we observed differential levels of NKp46^+^ cells. (Figure 3Aii, 3Aiii). In mice tissue samples, we found more NKp46^+^ cells in orthotopic tumors than in subcutaneous tumors (Figure 3B, 3C). RT-qPCR analysis of transcript levels of genes that encode cytokines or chemokines related to natural killer (NK) cell functions, we found higher expression of IL12, IL15, and IL18 in orthotopic tumors than subcutaneous tumors (Figure 3D). Flow cytometry analyses showed no difference in the number of dendritic cells (DCs) and macrophages between the two models (Figure 3E). We have carried out staining for CD80^+^ (M1 subtype) and CD206^+^ (M2 subtype) in both of our CRC models. Our data showed no significant difference in the proportion of the M1 and M2 subtypes (Supplementary Figure 2). However, we found more CD11b^+^, CD11c^-^, and Ly6C^+^ or Ly6G^+^ myeloid-derived suppressor cells (MDSCs) in subcutaneous tumors than orthotopic tumors (Figure 3F).

### Overall antitumor immune response increased in orthotopic tumors

**Figure 4:**
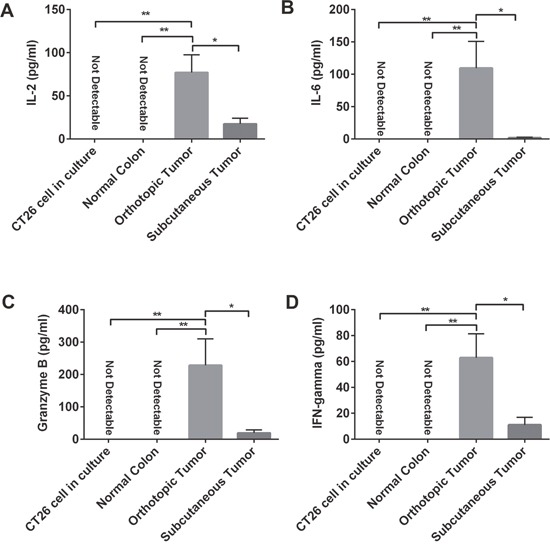
Expression of IL2, IL6, granzyme B, and IFNγ varied in orthotopic and subcutaneous tumors Using the enzyme-linked immunosorbent assay (ELISA) method, we measured the expression of some proinflammatory and cytotoxic cytokines, such as IL2 **(A)**, IL6 **(B)**, granzyme B **(C)**, and IFNγ **(D)**. In CT26 tumor cell culture and in normal colon tissue in BALB/c mice, those cytokines could not be detected. But in both orthotopic and subcutaneous tumors in mice, we detected expression of those cytokines: expression was higher in orthotopic tumors than in subcutaneous tumors. IFN-γ: interferon-gamma; IL: interleukin. *P < 0.05; ** *P* < 0.01.

Our analysis indicated a more robust immune cell presence in the orthotopic model. Further, we determined the overall inflammatory and antitumor immune response intensity by measuring the concentrations of IL6, IL2, IFNγ and granzyme B. We found that CT26 cells and normal colon tissues did not express IL6, IL2, IFNγ and granzyme B. However, compared with subcutaneous tumors, orthotopic tumors that grew in the colon microenvironment showed higher expression of all the cytokines tested (Figure 4). These findings support our observation that orthotopic tumors tend to have more antitumor immune cells and fewer immunosuppressive cells, suggesting that orthotropic tumors have a stronger overall antitumor immune response than subcutaneous tumors.

### Immune checkpoint expression and efficacy of ICBT differed between orthotopic and subcutaneous models

**Figure 5:**
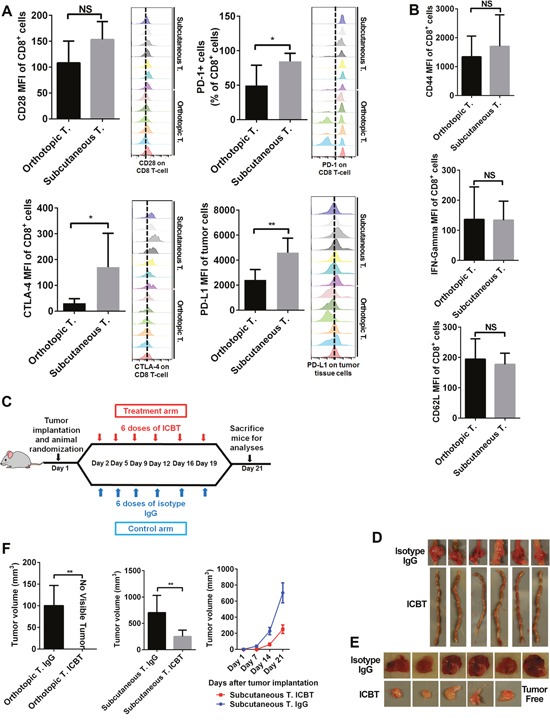
Immune checkpoint profile and response to ICBT differed in orthotopic and subcutaneous tumors Expression of inhibitory immune checkpoints PD1 and CLTA4 was higher on tumor-infiltrating T cells in subcutaneous tumors **(A)**. Expression of the stimulatory immune checkpoint CD28 was also higher on tumor-infiltrating T cells in subcutaneous tumors, but the difference was not statistically significant (P=0.057) **(A)**. Expression of PDL1 was higher in subcutaneous tumors **(A)**. Expression of T-cell activation/function markers (IFN-γ, CD62L, and CD44) was the same on tumor-infiltrating T cells in subcutaneous and orthotopic tumors **(B)**. To compare the drug sensitivity of the 2 models, we administered anti-CLTA-4 and anti-PD1 with a moderate intensity **(C)**. At the endpoint, orthotopic tumors showed a better response; they were thoroughly blocked by ICBT, whereas, the subcutaneous tumors can only be partly controlled by ICBT **(D-F)**. ICBT: immune checkpoint blockade therapy; IFN-γ: interferon-gamma; T: tumor. *P < 0.05; **P < 0.01.

Besides the number of immune cells, the activation status of cytotoxic immune cells is also critical in regulating antitumor immune response. The functional status of immune cells in tumors was determined by measuring the expression levels of immune checkpoints and T-cell activation markers. We generated orthotopic and subcutaneous CRC models by injecting same number (10^5^) of cells. In subcutaneous tumors, we observed higher levels of immune checkpoints such as CTLA4 and PD1 on T cells compared to orthotopic tumors (Figure 5A). Further, PDL1 expression was also higher in subcutaneous tumors than in orthotopic tumors (Figure 5A). However, we observed no significant differences in expression of T-cell activation markers such as CD62L, CD44, and IFNγ in tumor-infiltrating T cells in both tumor models (Figure 5B). We further investigated whether these two tumor models with different immune profiles show varying response to immune checkpoint blockade therapy (ICBT). Towards this, mice were either treated with IgG or anti-PD1 plus anti-CTLA4 in six doses for three weeks (Figure 5C). This treatment regime induced response in both orthotopic and subcutaneous tumors (Figure 5D, 5E, and 5F). After six doses of treatment, we did not observe any tumor growth in the orthotopic model (Figure 5D). However, in subcutaneous model, although the tumors responded to ICBT (Figure 5E and 5F, Supplementary Figure 3), residual tumors were still observed. To determine tumor vascularization that may have potential confounding effect on ICBT treatment, we stained CD31 in two of our CRC models. Our staining patterns suggest that no obvious difference of capillary density in these two models (Supplementary Figure 4).

## DISCUSSION

Studies in mice are frequently used to provide the rationale for testing anti-tumor therapies in phase 1 clinical trials [21]. The traditional approach, using human xenografts in immuno-compromised mice, is not amenable to testing immunomodulatory anti-tumor agents due to the absence of a physiological immune system. The mouse models of tumors, including human xenograft tumors and syngeneic tumors, were suitable for preclinical studies that tested cytotoxic drugs [21]. However, immunotherapy has a different mechanism for eliminating tumors: it kills tumor cells by enhancing or rebuilding the antitumor immune response in patients (rather than by directly killing tumor cells)[1]. The efficacy of immunotherapy, especially ICBT largely depends on the immune microenvironment of tumors [22, 23].

To maximize opportunities to translate novel immunotherapy strategies from preclinical studies to clinical application, CRC mouse models are needed that can mimic physiologically relevant microenvironment [11]. In this study, we analyzed two immune-competent models using murine CRC cell lines (CT26 and MC38) in their matched immune-competent hosts (BALB/c and C56BL/6). We demonstrated that these models can be used to test the efficacy of checkpoint blockade therapy, anti-CTLA4 and anti-PD1. Furthermore, we demonstrated that immune profiles and response are different in an orthotopic tumor microenvironment compared to subcutaneous tumors.

In line with previous studies, we observed human CRC subtypes with immune cells well- and poorly-infiltrated tumors [12, 19]. The immune cells poorly-infiltrated tumors are insensitive to ICBT [24], while the well -infiltrated tumors are more sensitive to ICBT [25]. Our investigation of adaptive immune profiles of orthotopic and subcutaneous tumors in mice represents a key step in mimicking different clinical settings in mouse models. The two CRC tumor models produced diverse immune phenotypes in terms of T cells infiltration. Orthotopic tumors were better infiltrated with T cells than subcutaneous tumors, same trend was also observed with B cells. Similar results were also observed in a cecum model [26], in which higher frequency of CD8^+^ T cells were detected in Peyer's patches in response to tumor challenge. Furthermore, studies have shown that regulatory T-cell (Treg) inhibits the function of cytotoxic T-cell and selectively kills antigen presenting cells including B cells [27]. However, in our study, the proportion of Tregs among all T cells was similar in orthotopic and subcutaneous CRC models (data not shown). This suggests that Treg infiltration is not affected by tumor location.

Innate immune cells, depending on their differentiation and functional status, either suppressed or promoted tumor formation [28]. NK-cell is the major cell type in innate immunity that have antitumor functions [29]. Enhancing the antitumor effects of NK cells via heterodimeric bispecific single chain variable fragments (scFv) killer engagers has been very promising in preclinical models [30]. In human CRC tissue samples, we observed a subset of samples with infiltrated NK cells. In mice, orthotopic tumors had more NK-cell infiltration than subcutaneous tumors. In a caecum model, higher levels of NK cells were founded in mesenteric lymph nodes (MLNs) of tumor group compared with MLNs of control groups [26]. Notably, subcutaneous tumors had more immunosuppressive cells such as MDSCs, compared to orthotopic tumors. On the other hand, we found the same proportion of antigen-presenting cells, such as DCs and macrophages in these two tumor models. In terms of expression of IL2, IL6, IFNγ and granzyme B, the orthotopic tumors showed relatively higher levels than subcutaneous tumors. Taken together, both adaptive and innate immune response are stronger in orthotopic model than in subcutaneous model. Similar to our observation, another study showed that tumors in the flank had poor immune response compared with tumors in the dorsal region of the foot [31]. On the other hand, the strong immune response observed in orthotopic tumors may explain why higher numbers of tumor cells are required to establish orthotopic CRC model than the subcutaneous tumors. Further studies are warranted to decipher the molecular mechanisms that regulate immune response in these models.

By comparing the two mouse models, we found that inhibitory immune checkpoint proteins, such as CTLA4, PD1, and PDL1 were expressed at lower levels in orthotopic tumors compared to subcutaneous tumors. Such checkpoints are significant factors in regulating activation of adaptive immune cells, especially T cells [32, 33]. We also saw a trend (p < 0.057) towards higher expression of the stimulatory immune checkpoint CD28 in subcutaneous tumors. These findings suggested that T cells in subcutaneous tumors were activated, but under strong suppression of immunosuppressive factors including immune checkpoint. These data were in line with our results that subcutaneous tumors have a weak immune response.

Response to ICBT in these two models was also investigated. Multiple ICBT protocols with large differences in treatment intensity have been reported in preclinical studies [34–36]. It also has been demonstrated that dual blockade of the PD1 and CTLA4 pathways increased the antitumor effects via enhancing immune effector cell/regulatory T-cell ratio in an animal model [37]. Considering that the CT26 tumors were relatively sensitive to ICBT [38], we administrated immune checkpoint blockades with a moderate intensity. This treatment plan would rule out false positive or negative effects due to inappropriate therapeutic dose. We found that tumors in both models responded to ICBT. But orthotopic tumors were more sensitive: they were totally blocked by ICBT. Subcutaneous tumors responded to ICBT, based on reduced growth; yet at the end of our study period, small subcutaneous tumors were still present. There was no difference observed in the CD31 levels between these two CRC tumor models suggesting that capillary density did not influence delivery of ICBT. Taking all data together, the subcutaneous model mimics a poor immune infiltrated and heavily immunosuppressive phenotype, whereas the orthotopic model can mimic relatively well-immune infiltrated CRC in a physiologically relevant microenvironment.

**Figure 6:**
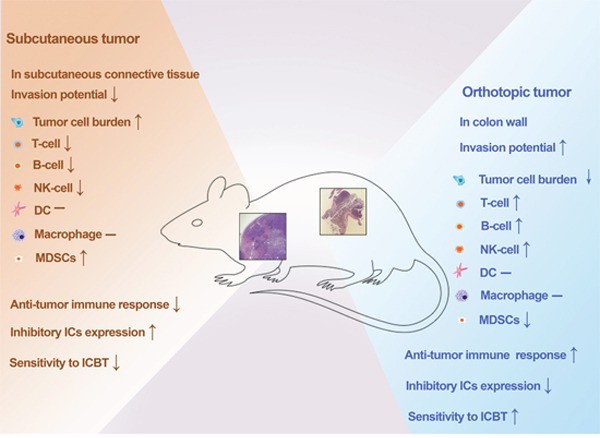
Summary of comparisons between the novel orthotopic CRC model and classic subcutaneous CRC model Orthotopic tumors required a higher number of tumor cells for tumorigenesis, but showed a stronger invasion ability, antitumor immune response, and sensitivity to ICBT. ICBT: immune checkpoint blockade therapy.

As a prerequisite for translating innovative immunotherapy strategies from bench to bedside, appropriate experimental CRC models are needed to accurately mimic different clinical settings. Our study investigated the key characteristics of a novel orthotopic CRC model, and showed significant differences between the orthotopic model and subcutaneous model in immune profiles and ICBT. Our study indicates that there remains a role for the subcutaneous model in immunotherapeutic studies, because they are easy to establish, are very stable, and they mimic a relatively sparse immune microenvironment. Our orthotopic model, on the other hand, provides another useful option, that better mimics CRC tumors with higher levels of immune infiltration in human patients (Figure 6). Taken together, our study identifies the influence of tumor location on immune response in CRC mouse models. Moreover, we highlight the significance of model selection in immunotherapy studies, and demonstrate a role for our novel endoscopy-guided orthotopic CRC model that could supplement current subcutaneous models to increase the translational potential of preclinical CRC immunotherapeutic studies.

## MATERIALS AND METHODS

### CRC cell lines

CT26, a CRC cell line generated from BABL/C mice, was purchased from the American Type Culture Collection (ATCC, Manassas, VA) and was cultured in RPMI-1640 medium with 10% fetal bovine serum (FBS) and 1% penicillin-streptomycin (pen-strep). MC38, a CRC cell line generated from C57BL/6 mice, was a gift from Dr. Nicholas Haining (Harvard University) and was cultured in Dulbecco's modified Eagle's medium (DMEM) with 10% FBS and 1% pen-strep. HT29, a human CRC line, was purchased from ATCC and cultured in McCoy's 5A medium with 10% FBS and 1% pen-strep. For *in vivo* imaging experiments, we generated a stably transfected CT26 cell line with firefly luciferase (CT26-Luc). Cell lines obtained from ATCC resource were authenticated by the vendor.

### Mice

BALB/c mice (6-8 weeks old, Jackson Laboratory, Bar Harbor, ME) were used for grafts using CT26 and CT26-Luc cells. C57/B6 mice (6-8 weeks old, Charles River Laboratories, Wilmington, MA) were used for MC38 grafts. Athymic nude mice (6-8 weeks old, Charles River Laboratories) were used for HT29 grafts. All mice were kept in a specific pathogen-free facility and had unrestricted access to water and food and a controlled 12-hr day-night cycle. Animal studies were approved by the institutional animal care and use committee of the University of Minnesota.

### Tumor implantation

To perform in *vivo* high-resolution colonoscopies, we used the Mainz Coloview mini-endoscopic system (Karl Storz Endoskope, Tuttlingen, Germany). For orthotopic tumor cell implantation in the colon wall, we anesthetized mice with ketamine (100 mg/kg) combined with xylazine (10 mg/kg) via intraperitoneal injection. To minimize colon movement, contraction, and secretion, we administered atropine (0.04 mg/kg, intraperitoneally). After tumor cell implantation, mice were put on a heating pad until fully recovered. When monitoring tumors, we anesthetized mice with Avertin (250 to 500 mg/kg, intraperitoneally) 5 minutes before the procedure, to minimize duration of anesthesia. For subcutaneous tumor cell implantation, we suspended tumor cells in a Matrigel matrix, then inoculated the suspension in the flank of legs.

### Treatment arm

After tumor cell implantation, mice were randomly separated into a treatment arm and a control arm. In the treatment arm, mice were injected with anti-mouse PD1 (Clone: RMP1-14, 10 mg/kg, twice per week) and anti-mouse CLTA4 (Clone: UC10-4F10-11, 5 mg/kg, twice per week) (both from BioCell Technology LLC, Newport Beach, CA). In the control arm, mice were injected with an IgG isotype (15 mg/kg, twice per week) (BioLegend, San Diego, CA).

### *In vivo* imaging

To monitor orthotopic CRC tumors established by CT26-Luc cells, we used the IVIS Spectrum in *vivo* imaging system (PerkinElmer, Waltham, MA); 10 minutes before imaging, we injected mice with D-Luciferin, GoldBio (150mg/kg, intraperitoneally) and then anesthetized them with isoflurane. For all mice, we set exposure time of imaging as 60 sec.

### Flow cytometry

Flow cytometry was used to measure immune cell infiltration and activation markers. Harvested tumor tissue was digested in a solution of collagenase IV (5 mg/ml) and deoxyribonuclease (DNase, 50 units/ml) at 37° C for 1 hr and filtered through a 40-μm cell strainer. Cells were centrifuged and resuspended in red blood cell lysis buffer for 10 minutes, followed by another round of centrifugation.

The following antibodies were purchased from BioLegend or BD Biosciences (San Jose, CA): CD3 (17A2), CD19 (6D5), CD4 (GK1.5), CD8 (53-6.7), CD11b (M1/70), CD11c (N418), CD28 (37.51), PD1 (29F.1A12), CTLA4 (UC10-4B9), PDL1 (10F.9G2), CD44 (IM7), CD62L (MEL-14), interferon-gamma (IFNγ, XMG1.2), Ly6C (HK1.4), Ly6G (1A8), F4/80 (BM8), and I-Ad (AMS-32.1). Cells were stained with surface marker antibodies first, and then fixed and permeabilized for staining with intracellular markers. Data was analyzed using FlowJo software (Tree Star, Inc., Ashland, OR).

### Histology and immunostaining

Immediately after mice were sacrificed, tumor tissue was fixed in 10% formalin before paraffin embedding. Standard procedures were used for hematoxylin and eosin (H&E) staining. For immunostaining, formalin-fixed, paraffin-embedded tissue was treated with xylene, rehydrated with ethanol, and heated in a microwave with citric buffer to retrieve antigens. For blocking purpose, the tissues were incubated for 30 minutes, with 5% bovine serum albumin buffer. Followed by overnight incubation at 4° C, with primary antibodies: anti-CD3 antibody, anti-CD4 antibody, and anti-NKp46 antibody (Abcam, Cambridge, United Kingdom) at 1:100 dilutions, anti-CD8 antibody (Novus Biologicals, Minneapolis, MN) at 1:20 dilution, anti-CD31 (Novus Biologicals, Minneapolis, MN) at 1:100, anti-F4/80 (R&D system Minneapolis, MN) at 1:100, anti-CD206 (R&D system Minneapolis, MN) at 1:100, as well as anti-CD80 (Novus Biologicals, Minneapolis, MN) at 1:100. After washing, tissues were incubated with fluorescence-conjugated secondary antibodies at room temperature for 1 hr. Slides were prepared with antifade mountant with 4’,6-diamidino-2-phenylindole (DAPI).

### RT-qPCR

The mirVana microRNA (miRNA) Isolation Kit was used to extract RNA. We used 500 ng of total RNA for real-time quantitative reverse-transcriptase polymerase chain reaction (RT-qPCR) analysis with the QuantiTect Reverse Transcription Kit (Qiagen, Hilden, Germany). To measure cDNA samples, we used the LightCycler 480 Instrument (Roche Life Science, Indianapolis, IN) normalized to 18S ribosomal RNA (rRNA) expression. Primer sequences are included in Supplementary Table 1.

### ELISA

ELISAs were performed to measure granzyme B, IFNγ, IL6 and IL2 using ELISA kits from Affymetrix (Santa Clara, CA) according to manufaturer's protocol. All samples were normalized based on protein concentrations measured using a BCA protein assay (Pierce Chemical Company, Dallas, TX).

### Statistical analysis

For all statistical analyses, we used GraphPad Prism 6.0 (GraphPad Software, San Diego, CA). To compare the treatment arm and the control arm, we used the Student *t* test. For multiple group data, we used the one-way analysis of variance (ANOVA) method; for multiple pairwise comparisons, we performed a Bonferroni post hoc adjustment. All data are plotted as the mean ± standard deviation (SD). Two-sided *P* values < 0.05 were considered statistically significant.

## SUPPLEMENTARY MATERIALS FIGURES AND TABLE




