# Supplementary material for: Tumor location impacts immune response in mouse models of colon cancer
Source: Oncotarget. 2017 Jun 9;8(33):54775–87. doi: 10.18632/oncotarget.18423 (PMC5589620; doi:10.18632/oncotarget.18423)
Supplement: Supplementary file 1 [file oncotarget-08-54775-s001.pdf]

Tumor location impacts immune response in mouse models of colon cancer

SUPPLEMENTARY MATERIALS

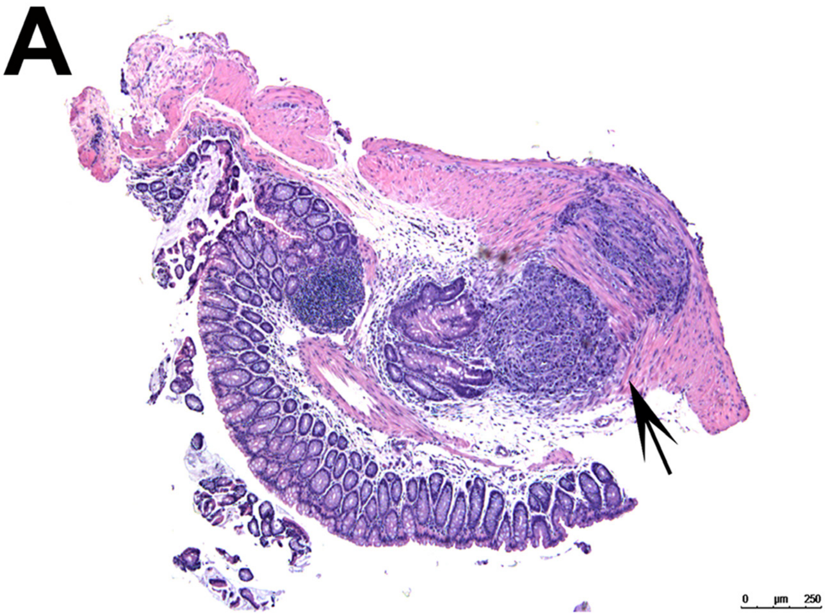

Supplementary Figure 1: Orthotopic tumors with human CRC cell line. The tumor model was established by injecting 10^5 HT29 cells in nude mice colon. H&E staining showed invasive tumor cells in the muscularis externa.

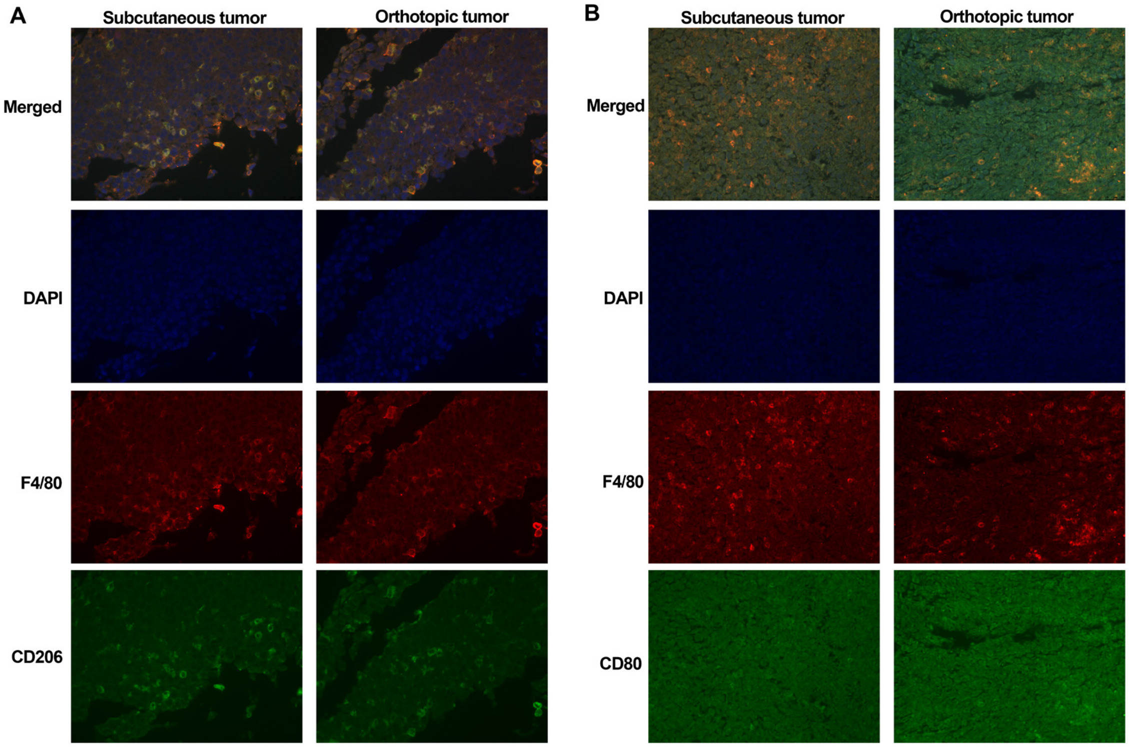

Supplementary Figure 2: Macrophage subtypes in the subcutaneous and orthotopic tumors. F4/80 and CD206 were co-stained in subcutaneous and orthotopic tumors. Most macrophages observed were M2 phenotype (F4/80$^+$, red) and (CD206$^+$, green) **(A)**, but not with M1 phenotype (CD80$^+$, green) **(B)**.

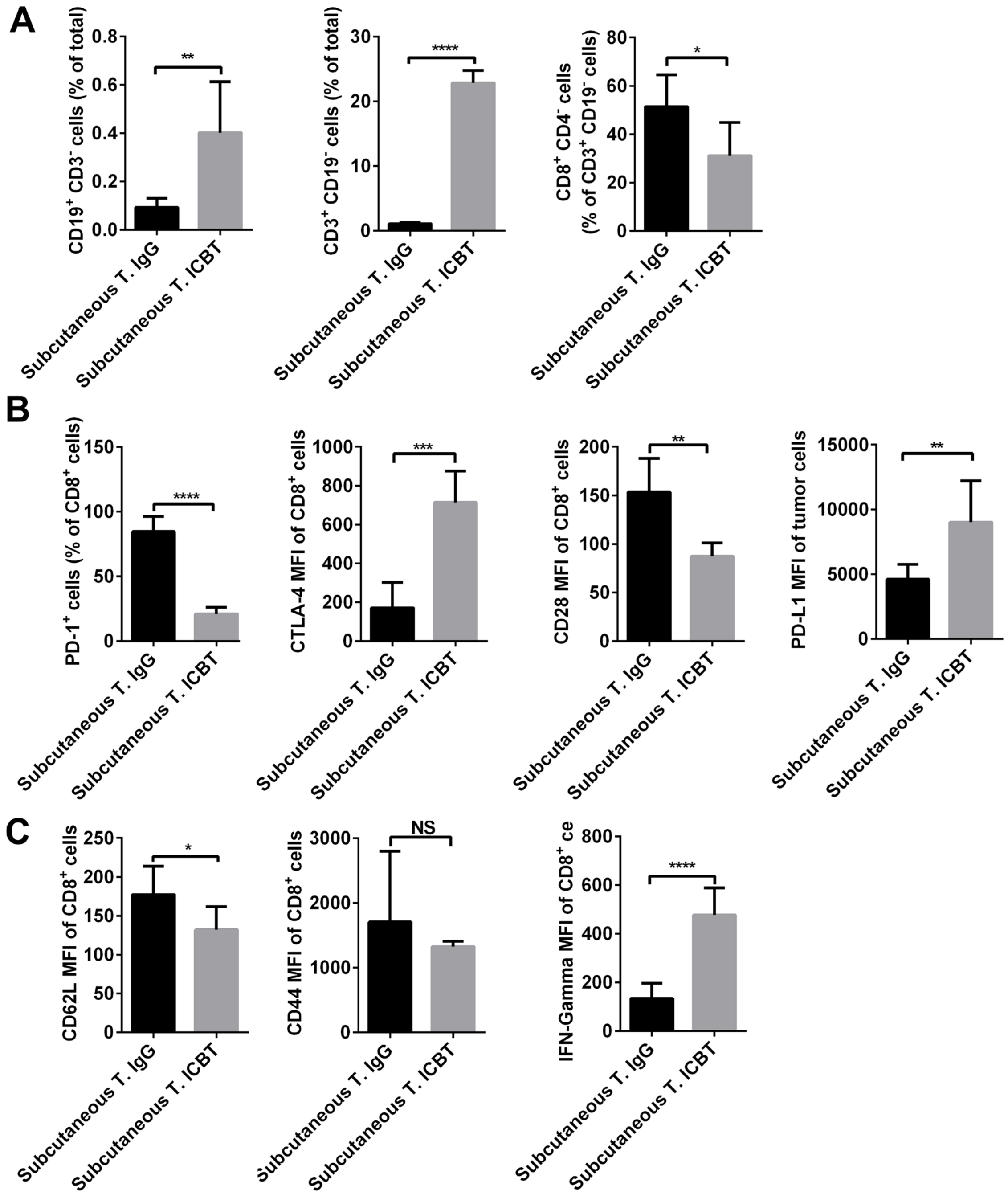

Supplementary Figure 3: Response of subcutaneous tumors to ICBT. After ICBT, the number of tumor-infiltrating B cells and T cells dramatically increased (**A**). But the proportion of CD8+ T cells decreased **(A)**. Expression of PD1 and CD28 on T cells was downregulated by ICBT; however, expression of CTLA4 on T cells and of PDL1 on tumor cells were upregulated **(B)**. Expression of CD62L on T cells decreased, and expression of IFNγ increased, indicating a more activated phenotype of tumor infiltrating T-cell after ICBT **(C)**. ICBT: immune checkpoint blockade therapy; IFN-γ: interferon-gamma; T: tumor. *P < 0.05; **P < 0.01; ***P < 0.001; ****P < 0.000.

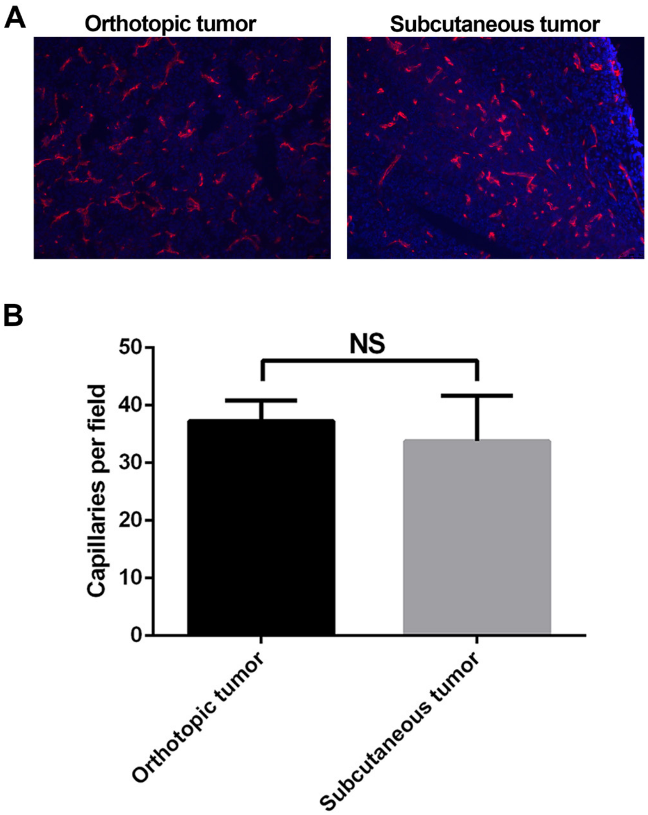

Supplementary Figure 4: CD31 staining in the subcutaneous and orthotopic tumors. Representative pictures of CD31 staining **(A)**. No significant difference was observed in CD31 positive cells **(B)**.

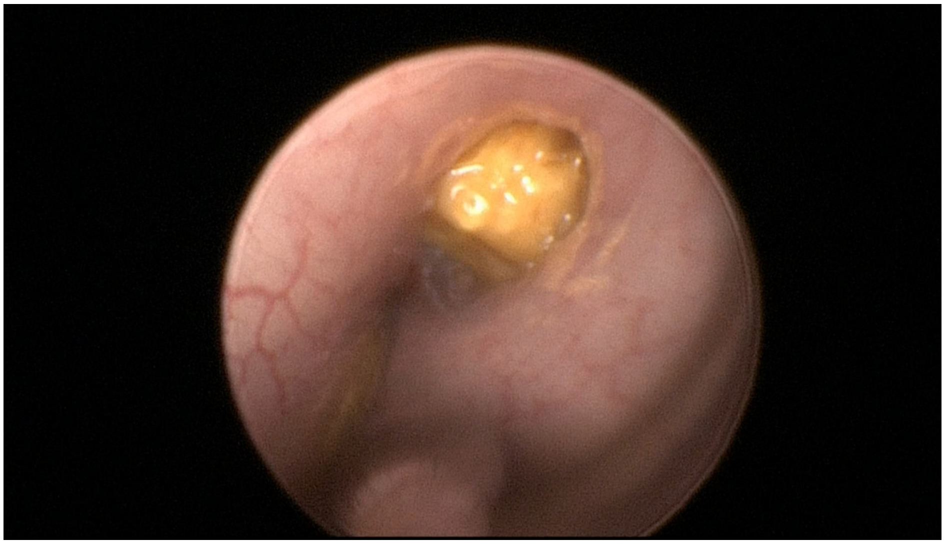

Supplementary Video 1: This video shows successful implantation, as confirmed by a clearly seen mucosal lifting sign, in a mouse anesthetized by ketamine and xylazine that also received atropine—a drug combination that resulted in best colonic relaxation and a more stable implantation. No leaking of injected cells were observed after tumor cell injection, indicating the high efficiency of tumor cell delivery of our method. To enhance engraftment, we recommend two operators for this procedure, with one person performing the endoscopic examination and the other performing the injection. We also found that the optimal needle diameter was 30-gauge.

See Supplementary Video 1

Supplementary Table 1: Primers used in the study

Gene name	Forward primer	Reverse primer
18SrRNA	GTTGGTTTTCGGAACTGAGG	AGTCGGCATCGTTTATGGTC
NCR1/NKp46	TTGCCAACTGAAGACTGCCA	TCCCTCTGTGAGCCCTAGTC
CD19	GTCATTGCAAGGTCAGCAGTG	GGGGTCAGTCATTCGCTTCC
CCL5	ATATGGCTCGGACACCACTC	ACTTGGCGGTTCCTTCGAG
IL-2	ATGAACTTGGACCTCTGCGG	GTCCACCACAGTTGCTGACT
IL-18	TCAGACAACTTTGGCCGACT	CAGTCTGGTCTGGGGTTCAC
IL-15	TCCCAGTTGCAAAGTTACTGC	TTCTCCTCCAGCTCCTCACA
IL-12	CCGGTCCAGCATGTGTCAAT	GGACTGGCTAAGACACCTGG
CXCL11	CTTATGTTCAAACAGGGGCG	TGCATTATGAGGCGAGCTT
CXCL10	GCTGCAACTGCATCCATATC	AGGAGCCCTTTTAGACCTTT
CXCL9	GAAGTCCGCTGTTCTTTTCC	TTGACTTCCGTTCTTCAGTGT
CXCR3	CCTGCATAGTTGTATGGGGT	ATATGGGGCATAGCAGTAGGC
